# Intentionality, Morality, and the Incest Taboo in Madagascar

**DOI:** 10.3389/fpsyg.2016.00494

**Published:** 2016-04-07

**Authors:** Paulo Sousa, Lauren Swiney

**Affiliations:** ^1^Institute of Cognition and Culture, Queen’s University BelfastBelfast, UK; ^2^University of WarwickCoventry, UK

**Keywords:** incest taboo, intentionality attributions, moral judgments, Madagascar

## Abstract

In a recent article (Astuti and Bloch, 2015), cognitive anthropologists Astuti and Bloch claim that the Malagasy are ambivalent as to whether considerations of intentionality are relevant to moral judgments concerning incest and its presumed catastrophic consequences: when making moral judgments about those who commit incest, the Malagasy take into account whether the incest is intentional or not, but, when making moral judgments relating to incest’s catastrophic consequences, they do not take intentionality into account. Astuti and Bloch explain the irrelevance of intentionality in terms of incest entailing such a fundamental attack on the transcendental social order that the Malagasy become dumbfounded and leave aside considerations of intentionality. Finally, they claim that a similar dumbfound reaction is what is involved in the moral dumbfounding concerning incest that social psychologist Jonathan Haidt has found in the US. In this article, we argue that (i) Astuti and Bloch are unclear about many aspects of their claims (in particular, about the moral judgments at stake), (ii) they do not provide sufficient evidence that considerations of intentionality are deemed irrelevant to moral judgments relating to incest’s presumed catastrophic consequences (and hence for the claim that the Malagasy are ambivalent), (iii) their hypothesis that conceiving of incest as an attack on the transcendental social renders considerations of intentionality irrelevant lacks coherence, and (iv) the extension of their explanatory account to the moral dumfounding of American students in Haidt’s well-known scenario of intentional incest is unwarranted.

## Introduction

There are distinct, though interconnected, questions surrounding the topic of incest (see, e.g., Lieberman et al., 2003; Fessler and Navarrete, 2004; Gutierrez and Giner-Sorolla, 2007; Royzman et al., 2008, 2009; Piazza and Sousa, 2014; Wolf, 2014). One is the issue of incest avoidance, which concerns why humans generally avoid having sexual relations with their close kin. Another is the issue of the incest taboo, which concerns why there is a widespread prohibition against having sex with close kin, a prohibition that in many cultural contexts extends to more distant, classificatory kin, and is highly correlated with corresponding marriage prohibitions. A third is the issue of the relationship between incest and moral psychology, which concerns the nature of moral judgments concerning incest.

In a recent article (Astuti and Bloch, 2015), cognitive anthropologists Astuti and Bloch address aspects of the second and third issues above, discussing the relationship between the incest taboo and morality among the Malagasy in Madagascar.^1^ They are particularly interested in the extent to which considerations of intentionality (i.e., whether an action is intentional or not) play a role in moral judgments about incest among the Malagasy, given that there is a widespread belief that committing incest leads to catastrophic consequences (e.g., crops fail, canoes overturn at sea, children die) even when incest is unintentional (i.e., when those committing incest are not aware that they are related as kin).^2^

Astuti and Bloch are leading researchers in the area of cognition and culture. In bringing the question of intentionality attributions into the discussion of moral judgments concerning incest, their article speaks to traditional issues in legal and cultural anthropology (e.g., Goldman, 1993; Rumsey and Robbins, 2008), to attribution research in social psychology (e.g., Shaver, 1985; Weiner, 1995; Alicke, 2000; Malle et al., 2014), and to recent cognitive and neuroscientific research on the relation between causal reasoning, theory of mind and moral judgments (e.g., Young et al., 2010a,b). In focusing on a specific type of transgression (i.e., incest), their article speaks to the important question in moral psychology concerning the extent to which moral judgments are uniform across different domains of transgressions (e.g., Young and Saxe, 2011; Gray et al., 2012; Haidt, 2013). Last but not least, in dealing with a “non-WEIRD” cultural context (Henrich et al., 2010) and drawing from their extended fieldwork research, their article speaks both theoretically and methodologically to the issue of which aspects of human cognition are universal or culturally specific. For all these reasons, their article is an important contribution that deserves the attention of anthropologists, psychologists and cognitive scientists more generally.

Astuti and Bloch make two sorts of claims. At a more descriptive level, they claim that the Malagasy demonstrate an unusual ambivalence about whether the distinction between intentional and unintentional actions affects their moral judgments concerning incest and its catastrophic consequences. On the one hand, similarly to the way they evaluate other transgressions, the Malagasy consider this distinction to be relevant: for example, a couple is not considered to be “at fault” if they committed the incest unknowingly (and thus unintentionally). On the other hand, when the Malagasy think about and deal with incest’s catastrophic consequences, the distinction is no longer relevant: everyone, including the innocent (i.e., people who did not commit incest, let alone commit it intentionally), is “punished” by these consequences and has a duty of reparation. According to Astuti and Bloch, the irrelevance of intentionality when the Malagasy think about incest’s catastrophic consequences is unusual because it goes against the widely held assumption that considerations of intentionality are a universal component of causal reasoning about wrongdoing.

At a more explanatory level, Astuti and Bloch (2015, p. 1) claim that the reason considerations of intentionality are deemed irrelevant to moral judgments when the Malagasy think about and deal with incest’s catastrophic consequences is that they envisage the attack on the transcendental social order that incest represents, and consequently become dumbfounded and indifferent to considerations of intentionality: “This [incest] entails such a fundamental attack on kinship and on the very basis of society that issues of intentionality and blame become irrelevant”. In addition, Astuti and Bloch claim that this explanation can shed light even on the dumbfounding reasoning concerning incest that social psychologist Jonathan Haidt and colleagues have found in the US (Haidt et al., 2000, unpublished; Haidt, 2001; Haidt and Hersh, 2001; but see Royzman et al., 2015, for a criticism).

In this article, we critically examine Astuti and Bloch’s claims. Much of our criticism comes from the fact that Astuti and Bloch are not clear enough about the various types of moral judgments invoked in their discussion. Thus, firstly, we provide a characterization of these different types of moral judgments and of our view of the relevance of intentionality considerations in each case. Secondly, we focus on Astuti and Bloch’s claim that the Malagasy demonstrate an unusual ambivalence in their moral judgments concerning incest and its catastrophic consequences, arguing that they do not provide clear evidence for this claim. Thirdly, we focus on Astuti and Bloch’s theoretical explanation, arguing that it lacks coherence and is not supported by beliefs about the incest taboo even in other cultural contexts in Madagascar. Moreover, we argue that their appeal to the notion of dumbfounding is unhelpful, and supports neither their explanation nor its generalization to the US context.

## Moral Judgments and Intentionality Considerations

Throughout their paper, Astuti and Bloch’s frame their discussion in terms of an inquiry related to moral judgments of wrongdoing, and in the context of what they take to be a widespread assumption that considerations of intentionality are relevant to these judgments. However, there are different moral judgments at stake in their discussion: judgments related to culpability, punishment and duty of reparation, as well as judgments of wrongdoing strictly speaking. Moreover, considerations of intentionality may not play the same role across these different judgments. In order to discuss the import of Astuti and Bloch’s claims about the relevance or irrelevance of intentionality considerations in the case of incest, we provide a brief characterization of how these moral judgments differ from each other and of the extent to which considerations of intentionality are relevant to each of them.

A judgment of wrongdoing, strictly speaking, corresponds simply to the judgment that an action is a normative transgression, that is, that an action has been performed when there is a norm forbidding the action (for the structure of deontic concepts related to this type of judgment, see Beller, 2001, 2008; Bender and Beller, 2003; Sousa, 2009; Sousa and Piazza, 2014).^3^ This judgment is *often* unrelated to considerations of intentionality, since ordinary norms forbidding an action may simply specify that the action is forbidden rather than specify that the action as intentional action is forbidden. In other words, the irrelevance of whether the forbidden action is intentional or not is already built into the understanding of the norm itself. For example, suppose that destroying someone else’s property is deemed forbidden (note that the verb “destroy” does not imply intentionality), and take the case of a person who hits someone else’s vase, breaking it. Given this norm, people will judge that the person did something wrong—breaking the vase is a transgression of the norm, whether the person did it intentionally or unintentionally.

While a judgment of wrongdoing is the evaluation of an *action*, a judgment of culpability is the evaluation of a *person* for her actions and the consequences of her actions: the person being judged culpable is attributed a blemish in their record as a person for what she has done and caused (Zimmerman, 1988; Sousa, 2009).^4^ A judgment of culpability normally presupposes a judgment of wrongdoing in that the person being judged culpable is supposed to have done something wrong. However, one can judge that an action is wrong without judging that the person is culpable. Take the example of a person breaking someone else’s vase purely accidentally. People in general would judge that the action is wrong but that the person is not culpable.^5^ Finally, it is important to note that the concept of culpability has a graded structure (one may attribute more or less culpability) and that one of the variables that calibrates the amount of culpability is related to considerations of intentionality—if, in the vase example, the person hitting the vase was seen as reckless, people would attribute at least some culpability.

Judgments concerning punishment and reparation may be subsumed under the category of judgments of liability, since they relate to the impositions that one may be liable to when one does something wrong—the suffering that one should be subjected to or the reparations that one should make in light of the wrongdoing (for a discussion of these concepts, see Jackendoff, 2007, chap. 10; Baumard, 2010; see also Graeber, 2014, for their relation with the broader issue of debt and the origin of money). Thus, judgments of liability presuppose a judgment of wrongdoing. In addition, the concept of liability has a graded structure related to the graded structure of the concept of culpability, and, thereby, related to considerations of intentionality too (i.e., more intentionality/culpability implicates liability to suffer more punishment and/or to make more reparations). Astuti and Bloch (2015, p. 2) provide an example of graded liability judgments related to considerations of intentionality, when they described to the Malagasy two scenarios of a person knocking over someone else’s bucket—in one case accidentally and in the other via a deliberate kick. While the Malagasy deem both persons to have a duty to replace the bucket should it be broken, liability judgments in the two cases differ by degree: in the accidental case, the person will merely say sorry and replace the bucket, while in the intentional case “a fight will ensue and the victim will take the perpetrator to the village assembly, where *a more serious punishment might be dispensed (e.g., a monetary compensation in addition to the replaced bucket.)”* (Astuti and Bloch, 2015, p. 2; the emphasis is ours).^6^

As this section has made clear, there are different types of moral judgments, and considerations of intentionality ordinarily do not play the same role across these different judgments. Considerations of intentionality are relatively less important to judgments of wrongdoing qua a normative transgression than to judgments of culpability and liability—whereas they are often irrelevant to judgments of wrongdoing qua normative transgression, they are fundamental to calculate the degree of culpability and liability, even if judgments of liability and culpability can sometimes be dissociated (e.g., as in the above Malagasy case where the person is seen as having a duty to replace the broken bucket even if the person is not seen as culpable because the breaking of the vase was purely accidental).

## On Malagasy’s Ambivalence

Astuti and Bloch seem to accept that when making judgments about the culpability of a couple for committing incest, the Malagasy take intentionality into account. They presented the Malagasy with a story about two adult siblings who had sex, but who, having been completely separated at birth, did not know they were related. They report that the Malagasy judge that the siblings are not “at fault” (i.e., are not to blame)^7^ for having committed incest because they could not have known that they were siblings. Moreover, they seem to acknowledge this explicitly:

Whether wrong doing is done intentionally or not can thus be taken into account even in the case of incest. But the point is that attributing blame is a quite different concern than imagining a world without any incest rule, where what is experienced as necessary for one’s collective existence as human beings in under threat. Attributing blame, in other words, is a quite different matter than dealing with *loza* [incest’s catastrophic consequences]. (Astuti and Bloch, 2015, p. 5)

(…) we have also shown that considerations of intentionality are present in the way people access culpability even in the case of incest (Astuti and Bloch, 2015, p. 5).

As the first passage above also indicates, Astuti and Bloch contrast judgments of culpability with the case of the Malagasy’s moral judgments concerning incest’s catastrophic consequences (*loza*), where they argue that intentionality is not taken into account. Indeed, Astuti and Bloch indicate throughout their article that the principal evidence concerning the irrelevance of intentionality comes from three beliefs (and related behaviors) concerning incest’s catastrophic consequences:

(i) the belief that incest leads to catastrophic consequences, whether the incest was intentional or not;
(ii) the belief that the catastrophic consequences affect everyone (or that punishment befalls on everyone), including the innocent;
(iii) the belief that everyone in the community, including the innocent, has a duty to repair the damages that may follow a case of incest.

We question whether Astuti and Bloch discussion of these three beliefs and related behaviors provide sufficient evidence that the Malagasy are ambivalent about intentionality in any unusual way.

The content of the first belief does not say anything direct about moral judgments—it just states that an act of incest causes catastrophic consequences regardless of intentionality. Throughout their paper, Astuti and Bloch frame their discussion in terms of an inquiry on “causal reasoning” related to wrongdoing, but appear to be conflating two types of claims—the claim that intentionality considerations (a type of causal consideration, given that intentions and beliefs are deemed to have a causal role in the production of actions) are seen by the Malagasy as irrelevant to *moral* judgments concerning incest and its catastrophic consequences, and the claim that intentionality considerations are seen by the Malagasy as irrelevant to *more circumscribed causal* judgments concerning the relation between an act of incest and catastrophic consequences. The content of the first belief (that incest causes catastrophic consequences regardless of intentionality) does confirm the claim that intentionality considerations are seen by the Malagasy as irrelevant to causal judgments concerning the act of incest and catastrophic consequences, but it does not provide any direct evidence for the claim that intentionality considerations are seen by the Malagasy as irrelevant to moral judgments.

We shall leave aside Astuti and Bloch claim that intentionality considerations are irrelevant to causal judgments concerning the act of incest and catastrophic consequences, since this claim is secondary to the topic of this paper, which is the relation between intentionality considerations and moral judgments. Moreover, there is nothing unusual in the fact that the Malagasy do not take into account intentionality in making causal judgments about the relation between the act of incest and catastrophic consequences—what may be unusual here is the belief in a causal link between incest and catastrophic consequences itself, rather than the belief that this causal link exists regardless of intentionality. One can see this point more clearly by noticing that, in general, intentionality considerations are not fundamental to causal judgments concerning the relation between actions and their consequences. Take the case of someone shooting a gun with the aim of killing a person and causing the death of the person, and the case of someone shooting a gun with the aim of breaking a bottle but accidentally causing the death of a person. We imagine people in general would say that in both cases the action of shooting the gun caused the death of a person, regardless of the mental states involved (for some caveats related to this point, see Lagnado and Channon, 2008).

Now, although the first belief above does not say anything direct about moral judgments, it strongly suggests that there is nothing specified about intentionality in the way the Malagasy conceptualize the incest taboo, since the presumed fact that incest causes catastrophic consequences independent of considerations of intentionality constitutes an explicit justification for the existence of the incest taboo, for considering that incest is wrong. Accordingly, when an act of incest is performed, whether intentionally or unintentionally, this should be quite sufficient for the Malagasy to judge that the incest is wrong—the norm (i.e., the incest taboo) is still in force and the action (i.e., the incest) is a transgression of the norm. Although Astuti and Bloch often frame their discussion in terms of judgments of wrongdoing, they do not discuss at all the relation between judgments of wrongdoing qua normative transgression and considerations of intentionality. So, there is nothing clear in their article suggesting that they would like to claim that the Malagasy do not take into account intentionality in making moral judgments of wrongdoing qua normative transgression. More importantly, given that judgments of wrongdoing are often unrelated to considerations of intentionality (as we claimed in the previous section), it would not be in any way unusual for considerations of intentionality to be irrelevant to the Malagasy’s judgments that incest is wrong.

Astuti and Bloch describe the content of the second belief above in two different ways: in terms of “catastrophic consequences affecting everyone” or in terms of “punishment befalling everyone.” The first description does not say anything about moral judgments—it just states that the Malagasy think that incest’s catastrophic consequences affect everyone. Therefore, it cannot be evidence for a claim about the irrelevance of intentionality considerations to moral judgments either. The second description (“punishment befalling everyone”) does indicate something about a moral judgment concerning *punishment*, and the content of third belief (“everyone has a duty to repair the damages”) does refer to a moral judgment concerning *duty of reparation*. We focus on this evidence.

The relevant content of the second belief relates to punishment. Their somewhat implicit argument for claiming that this content would indicate the irrelevance of intentionality considerations to liability judgments is this: if the Malagasy believe that punishment falls on everyone, including the innocent (i.e., on people who did not commit incest, let alone commit it intentionally), then intentionality judgments are irrelevant to liability judgments concerning punishment.

This argument hinges on the assumption that the Malagasy conceptualize the catastrophic consequences in terms of punishment. However, it is unclear whether Astuti and Bloch’s description of the content of the second belief in terms of punishment (instead of simply in terms of causation) is a faithful translation of the way the Malagasy themselves conceptualize the catastrophic consequences, or whether it is a theory-laden redescription by the anthropologists.

The cross-cultural evidence concerning beliefs about a causal relation between incest transgressions and apparently unrelated harmful consequences such as crops failing suggests that this counter-intuitive casual relation may be understood in three distinct ways (cf. Wolf, 2014). The first one is in terms of supernatural agency punishment. In this instance, a supernatural agent (e.g., spirits, gods, God) is supposed to have caused the catastrophic consequence as punishment for the incest transgression. The second one is in terms of automatic punishment. In this instance, the catastrophic consequence is still understood as punishment for the incest transgression, but no supernatural agent is postulated to mediate the counter-intuitive causal relation. The third one is in terms of intrinsic natural consequence. In this instance, the catastrophic consequence is not understood as a punishment at all, but rather as a ‘natural’ disaster that is as an intrinsic natural consequence of the incest transgression.

Now, when Astuti and Bloch mention how the Malagasy discuss incest and its catastrophic consequences, they do not mention anything about the Malagasy discussing the catastrophic consequences in terms of punishment, involving supernatural agency or otherwise. Instead they emphasize an intrinsic mechanistic link between incest and the catastrophic consequences that is present even in the semantics of the verb used to describe an act of incest:

The word that Malagasy adults will almost certainly always use when discussing incest and contemplating its effects, is *loza*. The dictionary definition of this term is “calamity” or “disaster”; the verb for committing incest (*mandoza*) thus literally translates as causing a calamity or disaster (Astuti and Bloch, 2015, p. 3).

Indeed, the fact that the verb used to describe incest works like a lexical causative in which the feature *catastrophic consequences* (i.e., disaster) is part of the semantics of the verb counts against any punishment interpretation, and favors the intrinsic-natural-consequence hypothesis. To make a pertinent analogy, to interpret “loza” in terms of punishment would be like saying that the feature death, qua a semantic component of the lexical causative “to kill” (to cause death), can be interpreted as punishment for the behavior that lead to the death. In other words, if the feature *catastrophic consequences* (“loza”) is seen as an intrinsic component of incest as an action (“mandoza”), it cannot be seen as punishment for a component of the action.^8^

Of course, it is still possible that the Malagasy have different interpretations of the relation between incest and its catastrophic consequences, one of which may be in terms of these consequences being punishment for incest, given that beliefs about a counter-intuitive causal relation between an act of incest and catastrophic consequences constitute a typical reflective belief with a semi-propositional content that is susceptible to various interpretations by the believers themselves (Sperber, 1985, 1997). However, even under this interpretation, additional evidence would have to be provided to show the irrelevance of intentionality judgments to liability judgments in this respect. The fact that punishment is seen as befalling everyone does not show that the Malagasy think that punishment befalls everyone *equally*. It may be that, in cases of intentional incest, the Malagasy have intuitions that the catastrophic consequences qua punishment will affect those who committed incest (or those closely associated to them) more than the rest. More importantly, it may be that the Malagasy have intuitions that the catastrophic consequences qua punishment will be more extreme when incest is committed intentionally rather than unintentionally. No evidence concerning these points is provided in Astuti and Bloch’s article.

Let’s turn to the content of the third belief—everyone in the community has a duty to repair the damages that may follow a case of incest. Astuti and Bloch seem to appeal to a similar argument to demonstrate the irrelevance of intentionality considerations to judgments to liability: if the Malagasy believe that everyone has a duty to make reparation, even the innocent (i.e., those who did not commit incest, let alone commit it intentionally), then intentionality judgments are irrelevant to liability judgments.

Again, it is not clear to us whether the description of the content of this belief in terms of a duty of reparation is a faithful translation of the way the Malagasy themselves conceptualize the issue, or is a theory-laden redescription by the anthropologists. Alternatively, and consistent with the alternative hypothesis we put forward above about the Malagasy’s understanding of the catastrophic consequences in terms of intrinsic natural consequences, this belief may indicate simply a duty of mutual help in situations of natural catastrophes that have occurred or are envisaged to occur. Just think about the occurrence of natural disasters like a hurricane in a Western cultural context (leaving aside possible interpretations in terms of supernatural mediation), where people in the country feel the duty to help those afflicted by the catastrophe; or think about the mutual help involved in preventing the damages of a potential natural disaster.

Moreover, even if the Malagasy also think in terms of duty of reparation literally, additional evidence would have to be provided to show the irrelevance of considerations of intentionality to liability judgments. Again, the fact that the Malagasy think that everyone has a duty to repair does not show that they think that everyone has an *equal* duty of reparation. It may be that, in cases of intentional incest, the Malagasy believe those who committed incest (or those closely associated to them) should provide greater reparations than the rest for the disasters that have occurred or may be envisaged to occur. More importantly, it may be that the Malagasy have intuitions that the duty to repair would be more extreme in relation to those who commit incest intentionally than to those who commit incest unintentionally. No evidence concerning these issues is provided by Astuti and Bloch’s article. In fact, Astuti and Bloch do not provide any detailed evidence at all about what is involved in Malagasy’s reparation behavior concerning incest. The most they say is that “… a large number of innocent people are responsible for undertaking the difficult (expensive, dangerous, stressful) ritual work that is required to repair the damage and put things right again” (Astuti and Bloch, 2015, p. 2).

In sum, if the Malagasy conceptualize incest’s catastrophic consequences merely in terms of intrinsic natural consequences, then arguably their beliefs concerning these consequences are unrelated to moral judgments of punishment or duty of reparation. Even if we assume that the Malagasy *do* conceptualize the catastrophic consequences in terms that imply judgments of liability, the claim that intentionality considerations are deemed irrelevant to these judgments is not supported by the evidence that Astuti and Bloch provide. Thus, Astuti and Bloch’s article falls short of providing evidence for their claim that the Malagasy do not take into account intentionality in making judgments of liability concerning incest, and hence falls short of providing evidence for any unusual ambivalence.

## On Astuti and Bloch’s Explanatory Claims

Having posited an ambivalence in Malagay’s thinking, Astuti and Bloch go on to provide an explanation of why the Malagasy are ambivalent. Their explanation appeals to the transcendental nature that they presume humans attribute to social roles and norms: “they survive their incumbents; they extend beyond the life cycle, the frailty, the shortcomings of any one individual that inhabits them” (Astuti and Bloch, 2015, p. 3). For the Malagasy, they argue, kinship and its roles are experienced as this form of transcendental sociality, which “provides an image, however, vague, of a stable and lasting order and seems to afford certainty about what people ought to do and how they should behave—as mothers and fathers, as children and grandchildren” (Astuti and Bloch, 2015, p. 4). In this context, incest “invite[s] the thought that the rules we live by may be just flimsy fictions,” and thus constitutes a “total attack on the social” and a threat to “the transcendental in its entirety” (Astuti and Bloch, 2015, p. 4). According to Astuti and Bloch, because incest involves such an enormous breach of the transcendental social order, the Malagasy become dumbfounded and neglect considerations of intentionality in forming moral judgments.

Of course, we do not think that the presumed neglect of intentionality in forming moral judgments really requires an explanation, for, as we argued in the previous section, no clear evidence has been provided for the existence of an unusual ambivalence. But even if evidence were presented to support the descriptive claim, we would still have problems with their explanation.

Firstly, it is difficult to see why the fact that the Malagasy think about incest as a threat to the social order, interpreted as transcendental or not, would lead to indifference concerning whether a case of incest is intentional or not. Other things being equal, intentional transgressions are much more threatening to the social order than unintentional transgressions, for they are an index of willingness to bypass the social order and, possibly, of advocation of social change, whereas unintentional transgressions do not have such implications. Hence, the hypothesized link between thinking about incest’s catastrophic consequences and thinking about incest’s attack on the transcendental social order does not help explain the presumed irrelevance of intentionality judgments.

Secondly, the nature of this hypothesized link is unclear. Astuti and Bloch seem to be adopting some version of the well-known Durkheimian symbolist hypothesis (Durkheim, 1965; Skorupski, 1976), in which apparently irrational statements, like “incest causes catastrophes,” are to be interpreted as statements about society: “In ethnographic terms, as we have seen, incest is said to cause *loza*: calamity and disaster. In more abstract and theoretical terms, we now propose, incest is perceived as a threat to the very fabric of human sociality.” (Astuti and Bloch, 2015, p. 4). But the proposal that there is a symbolic link between the idea that incest causes *loza* and the idea that incest is an attack to the very fabric of society (the former being a symbol of the latter) is not without problems. It seems to be inconsistent with Astuti and Bloch’s own suggestion that the Malagasy understand the catastrophic consequences in terms of punishment for incest (see previous section), as it would entail that the Malagasy understand incest’s catastrophic consequences both as punishment, which supports the social order, and as the destruction of the social order. Moreover, as with most symbolist hypotheses a la Durkheim, this hypothesis explains neither why the Malagasy would use such an indirect mode of expression (why not talk directly about destruction of society instead of *loza*?), nor the fact that Malagasy’s behaviors seem compatible with an interpretation of loza simply in terms of intrinsic catastrophic consequences. Finally, the usual move from those who adopt a symbolist hypothesis — claiming that the symbolism is esoteric (the natives try to hide the real meanings from the anthropologists or outsiders) or unconscious (the real meanings are hidden from the natives themselves) — would create more problems than solve them (see Sperber, 1975).

Thirdly, although Astuti and Bloch may not want to extend their explanation to Madagascar generally (see footnote 1), it is worth pointing out that their explanation is not consistent with other cultural traditions in Madagascar. Huntington (1978) indicates that, among the Bara of southern Madagascar, the belief is that incest’s harmful consequences affect only the incestuous couple and their offspring. It is difficult to see how these restricted harmful consequences could be seen as symbolic of destruction of the social order. Also, Middleton (2002) indicates that, among the Karembola of southern Madagascar, who believe that incest has generalized catastrophic consequences as discussed by Astuti and Bloch, incest is not seen unambiguously as negative. She says:

For the Karembola, then, the morality of incest is highly ambiguous. It is not, as among the Merina of the Highlands of Madagascar, the ‘conceptual antithesis to kinship,’ ‘the ultimate wrong’ while kinship is the ultimate right (Bloch, 1971, p. 67). Rather, like other forms of taboo breaking, incestuous activity releases a fearsome power (*asy*) that can be turned by sacrifice into good or bad. Incest can easily render cattle sterile, cause women to bear ‘creatures’ (*biby*), harvests to fail, and people to die; but, if handled well, it can make people *masine* (‘blessed,’ ‘efficacious,’ ‘fertile’). Its power lies in its essential indeterminacy (Middleton, 2002, p. 203).

If, among the Karembola, kinship is not the ultimate parameter of social order and incest has such an ambiguity, it is difficult to suppose that the relation between incest and catastrophic consequences is conceived as an attack to the social order.

A final problem with Astuti and Bloch’s explanation is its incorporation of a notion of dumbfounding, which, in general terms, refers to a person being *unable* to explain why she makes a specific judgment (and being somewhat perplexed by this inability).

One issue is that Astuti and Bloch claim that considerations of intentionality become irrelevant *because the Malagasy are dumbfounded* by the attack on the transcendental social order that incest represents, but without providing any clear characterization of the connection between being dumbfounded and neglecting considerations of intentionality. They say:

“(…) the consequences of incest are indeed understood as catastrophic (…). And yet, when asked why this is so, our Malagasy interlocutors are stumped—or dumbfounded, to use a term used in the psychological literature on moral reasoning (…) they are unable to give a single and sufficient account of the relationship between cause (the breach of the taboo) and effect (*loza*) (Astuti and Bloch, 2015, p. 3).

However, it is unclear to us why being unable to give an account of the relationship between incest and its catastrophic consequences should impact the relevance of intentionality considerations. It is also unclear to us why Astuti and Bloch need to posit that this dumfounding is the result of the perception of a threat to the transcendental social order. We would argue instead that the Malagasy’s inability to provide a detailed causal account of the relationship between incest and its catastrophic consequences corresponds simply to the well-known fact that people normally do not have plain and systematic ideas about how counter-intuitive causation works.

Another issue is that Astuti and Bloch make an unconvincing analogy between their take on dumbfounding and the notion of moral dumbfounding in the context of Jonathan Haidt’s research (see aforementioned references). “Moral dumbfounding” in the context of Haidt’s research refers to the fact that Americans seem unable to provide an adequate explanation for their judgment that intentional incest is still wrong once the usual Western objections to intentional incest are counteracted (e.g., the couple uses precautions against pregnancy and acts in secrecy to avoid offending other people). Before explicating Astuti and Bloch’s analogy, it is important to emphasize that Haidt’s research is about people being unable to provide an adequate explanation for their judgments of *wrongdoing*, in the sense of judgments that an action is a normative transgression; the research is not about asking people to provide explanations for any judgments of liability. It is also worth noting that Haidt’s research concerns only cases of intentional incest and is unrelated to the question about the relevance of considerations of intentionality.

Turning to the dumbfounding analogy, Astuti and Bloch claim, on the one hand, that, like the American students in Haidt’s research, the Malagasy would evince dumbfounding in response to questions about wrongdoing in the context of Haidt’s scenario of intentional incest. But, as Astuti and Bloch seem to acknowledge in passing, the Malagasy would not have any problem in explaining why intentional incest is wrong if confronted with Haidt’s scenario: incest causes catastrophic consequences even if the aforementioned Western objections were counteracted.

On the other hand, Astuti and Bloch also suggest that American students’ moral dumfounding can be similarly explained in terms of the perception of an attack on the transcendental social order: “Their dumbfoundedness signals that what they are thinking and care about is the need to align themselves, jointly with others, with what, ultimately and fundamentally, makes people, namely, the transcendental” (Astuti and Bloch, 2015, p. 6). This is certainly an intriguing hypothesis, and they posit it as an alternative to Haidt’s social intuitionist account, which holds that moral dumfounding is the result of intuitive “gut feelings” that belie *post hoc* rationalization. However, we find the rationale of their explanation even less persuasive here than in the case of the Malagasy: principles of kinship are much less socially important in individualist societies like the US, and there is no widespread belief in the US context that incest has catastrophic consequences.

## Conclusion

Astuti and Bloch are some of the few cognitive anthropologists who combine long-term fieldwork involving participant observation and other qualitative methods with the more controlled tasks deployed by experimental psychologists and cognitive scientists, and we commend them in their perspective and the important contributions they have made. In their recent paper, they claim that their fieldwork has uncovered an unusual pattern of reasoning when the Malagasy think about the catastrophic consequences that are supposed to follow from breaking the incest taboo, and propose an explanatory account that can both explain this finding and potentially provide an account of the moral dumbfounding evinced by American students when they are asked to explain why intentional incest is wrong.

We have argued that their article falls short of providing evidence for their descriptive claims and that there are many aspects of their explanation that are unconvincing. We have also argued that many aspects of their central claims are unclear. One of our aims is to push them to address our concerns by developing a more detailed combined methodology and a more rigorous theoretical framework in relation to the current topic, as they have previously pursued in relation to other topics (see, e.g., Bloch et al., 2001; Astuti et al., 2004; Astuti and Harris, 2008). It is our hope that our article will instigate them to do so, as this would surely advance our knowledge on the relationship between considerations of intentionality, moral judgments and the incest taboo.

## Author Contributions

The primary theoretical arguments of the article were jointly developed by PS and LS. PS took the lead both in terms of theoretical framing and writing, while LS contributed substantially to these aspects.

## Conflict of Interest Statement

The authors declare that the research was conducted in the absence of any commercial or financial relationships that could be construed as a potential conflict of interest.

## Footnotes

1 Astuti and Bloch have pursued long-term ethnographic research among the Vezo on Western coast (Astuti), and among the Merina and the Zafimaniry of the central highlands (Bloch). Following them, we use the expression “people in Madagascar” (or “the Malagasy”) to refer to adult members of *these groups*, which, according to them, hold a similar view on the topic discussed in their paper. Later in the article, we shall bring into our discussion some evidence from groups from southern Madagascar (the Bara and the Karembola) to make a critical contrast, but without supposing that Astuti and Bloch wanted to extend their discussion and hypotheses to these other groups.

2 Across Madagascar the incest taboo varies in terms of the range of tabooed kin relations—e.g., in some parts, children of two brothers are tabooed, in other parts, they are not.

3 In this discussion, *moral* norms and wrongdoings are more specifically characterized a la the Turiel tradition, that is, in terms of a type of normative conviction that entails the sense that a transgression is ‘inherently’ wrong (see Astuti, 2007; Sousa and Piazza, 2014). This approach to specifying moral norms and wrongdoings, which is based on criteria such authority independence (moral norms/wrongdoings are seen as authority independent while conventional norms are not), is completely independent of how ordinary people would use the term “moral” to categorize norms or wrongdoings.

4 Note that there is the private judgment of culpability, which leads one to update the ‘moral file’ of the person being judged, and there is the public expression and communication of this judgment, which leads to the social construction of the person’s moral reputation (Sperber and Baumard, 2012). Note also that judgments of culpability do not correspond to judgments of moral character, although, arguably, there is a two-way street between these judgments (cf. Pizarro and Tannenbaum, 2011).

5 There is some evidence that a non-trivial proportion of individuals take into account intentionality (in terms of mental states such as belief and desire) when making judgments of wrongdoing/impermissibility (see Cushman, 2008), which apparently goes against both our claim above that considerations of intentionality are often irrelevant to judgments of wrongdoing (or, equivalently, to judgments of impermissibility) and our claim here that the judgment that an action is wrong/impermissible may be detached from the judgment that the agent is culpable in cases of pure accidents. This is not incompatible with our picture, as we are not claiming that considerations of intentionality are in general irrelevant to judgments of wrongdoing. The understanding of norms may vary across norms and individuals in terms of whether what is forbidden is understood more specifically as an intentional action or not (This variation in understanding also relates to the level of institutionalization of norms, with the legal system tending to explicitly build culpability qua levels of intentionality into the definition of crimes—see American Model Penal Code, for example.). Moreover, there is good evidence that many people engage in “excuse validation” (i.e., they hesitate to say that a purely accidental action is wrong/impermissible because they don’t want to suggest that the agent is culpable—see Turri and Blouw, 2015), which we interpret in terms of a pragmatic-communicative bias that is not fundamental to the conceptual structure related to judgments of wrongdoing.

6 Some anthropologists claim that various cultural traditions adhere to a notion of strict or absolute liability where considerations of intentionality are generally irrelevant to liability judgments. However, these claims are somewhat confusing and the evidence is actually weak (see Goldman, 1993; Sousa and Manoharan, 2016; but see Barrett et al., 2016). Although Astuti and Bloch allude to this type of anthropological claim in the beginning of their article, they clearly reject the idea that the Malagasy would constitute such a cultural tradition.

7 We take Astuti and Bloch’s translation in terms of the expression “at fault (for)” to correspond to the culpability sense of “is to blame (for).” For a detailed discussion of the different ordinary and technical meanings of expressions or words such as “at fault,” “blame,” “culpability,” “responsibility,” “accountability,” and “liability,” see Sousa (2009).

8 It is also worth noting that, if the catastrophic consequences are understood merely as intrinsic, natural causal consequences, this would easily explain why the Malagasy believe that incest has catastrophic consequences regardless of intentionality (the first belief described above).
